# Supplementary material for: Optimization Method for Phenolic Compounds Extraction from Medicinal Plant (Juniperus procera) and Phytochemicals Screening
Source: Molecules. 2021 Dec 9;26(24):7454. doi: 10.3390/molecules26247454 (PMC8708409; doi:10.3390/molecules26247454)
Supplement: Supplementary file 1 [file molecules-26-07454-s001.zip › molecules-1497913-supplementary.pdf]

Optimization method for phenolic compounds extraction from medicinal plant (*Juniperus procera)* and phytochemicals screening

Abdalrhaman M. Salih[1*], Fahad Al-Qurainy[1], Mohammad Nadeem[1], Mohamed Tarroum[1], Salim Khan[1] Hassan O. Shaikhaldein[1], Abdulrahman Al-hashimi[1], and Alanoud Alfagham[1] & Jawaher Alkahtani[1]

[1] Botany and Microbiology Department, College of Science King Saud University, P. O. BOX 2455, Riyadh 11451, Saudi Arabia

* Correspondence: abdalrahamanm@gmail.com

Abdalrhaman M. Salih has contributed significantly to this work

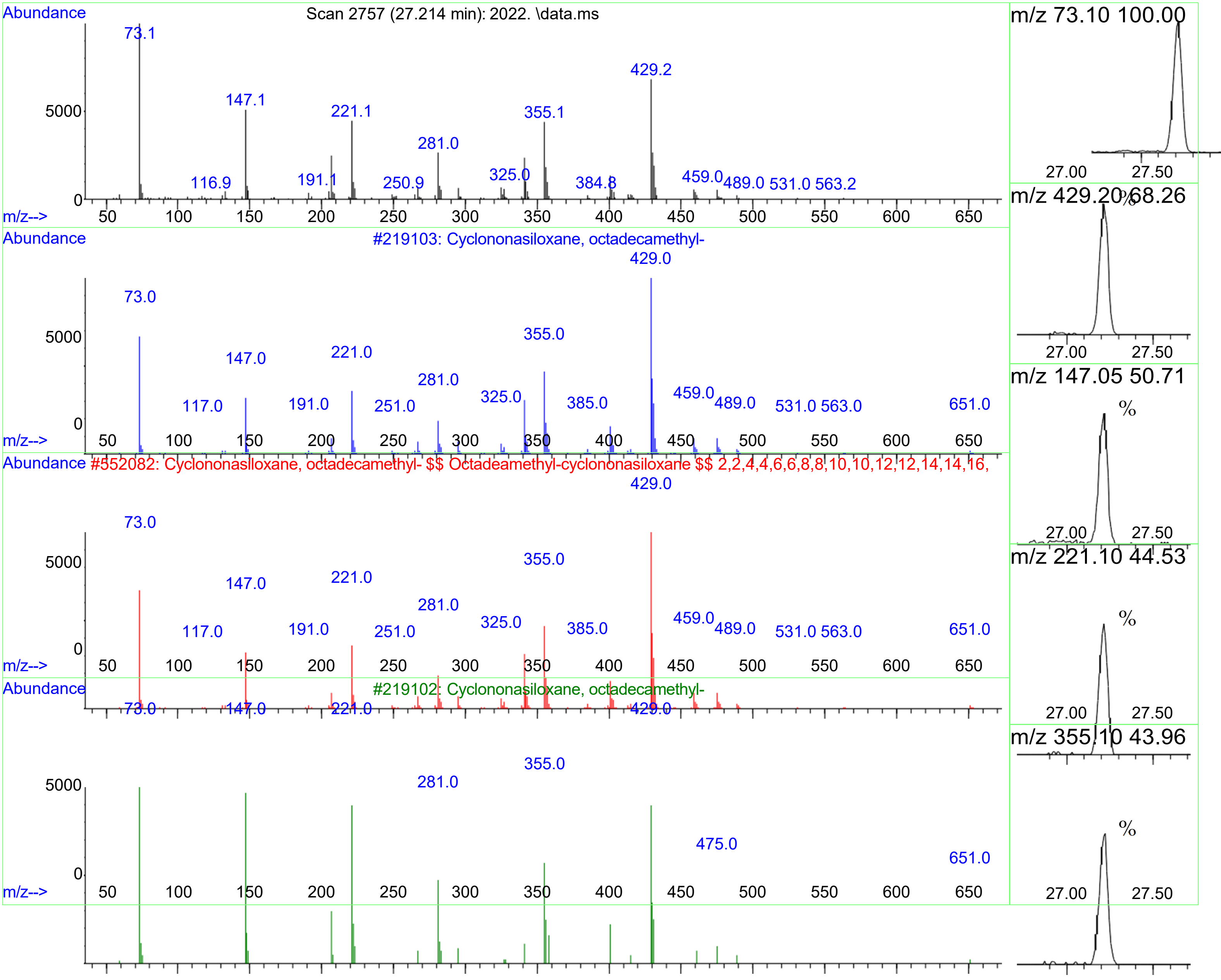

Oct 28 08:03:16 2020 Page: 2

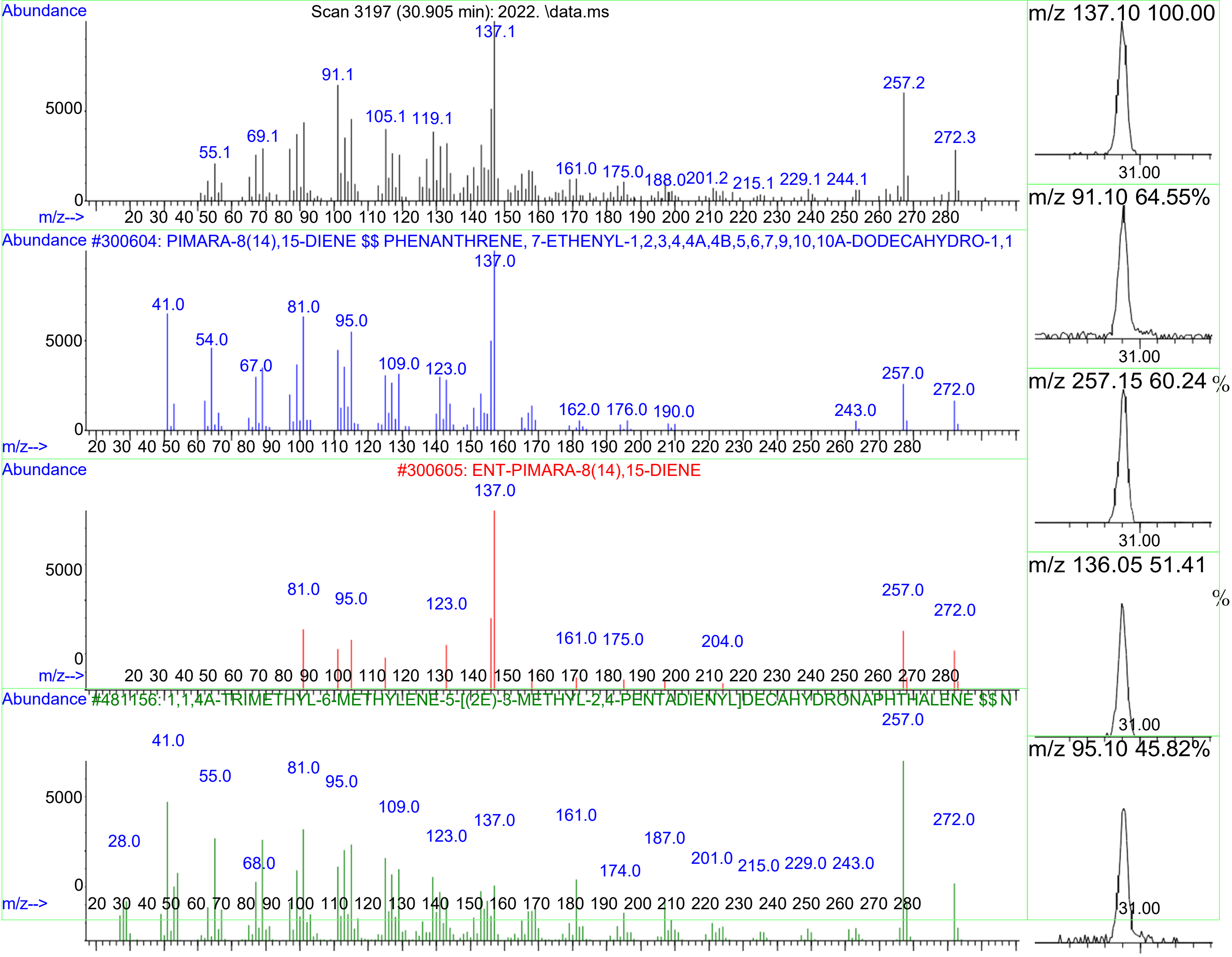
Abundance
Scan 3197 (30.905 min): 2022. \data.ms
137.1
91.1
257.2
5000
105.1 119.1
69.1
272.3
55.1
161.0 175.0 188.0 201.2 215.1 229.1 244.1
0
m/z-->
m/z 137.10 100.00
31.00
m/z 91.10 64.55%
31.00
m/z 257.15 60.24 %
31.00
m/z 136.05 51.41 %
31.00
m/z 95.10 45.82%
31.00
Abundance #300604: PIMARA-8(14),15-DIENE $$ PHENANTHRENE, 7-ETHENYL-1,2,3,4,4A,4B,5,6,7,9,10,10A-DODECAHYDRO-1,1
137.0
41.0
81.0
95.0
54.0
67.0
109.0 123.0
257.0
272.0
162.0 176.0 190.0
243.0
Abundance
#300605: ENT-PIMARA-8(14),15-DIENE
137.0
81.0
95.0
123.0
257.0
272.0
161.0 175.0
204.0
Abundance #481156: 1,1,4A-TRIMETHYL-6-METHYLENE-5-[(2E)-3-METHYL-2,4-PENTADIENYL]DECAHYDRONAPHTHALENE $$ N
257.0
41.0
55.0
81.0
95.0
109.0
137.0
161.0
28.0
123.0
187.0
272.0
68.0
201.0
215.0 229.0 243.0
174.0

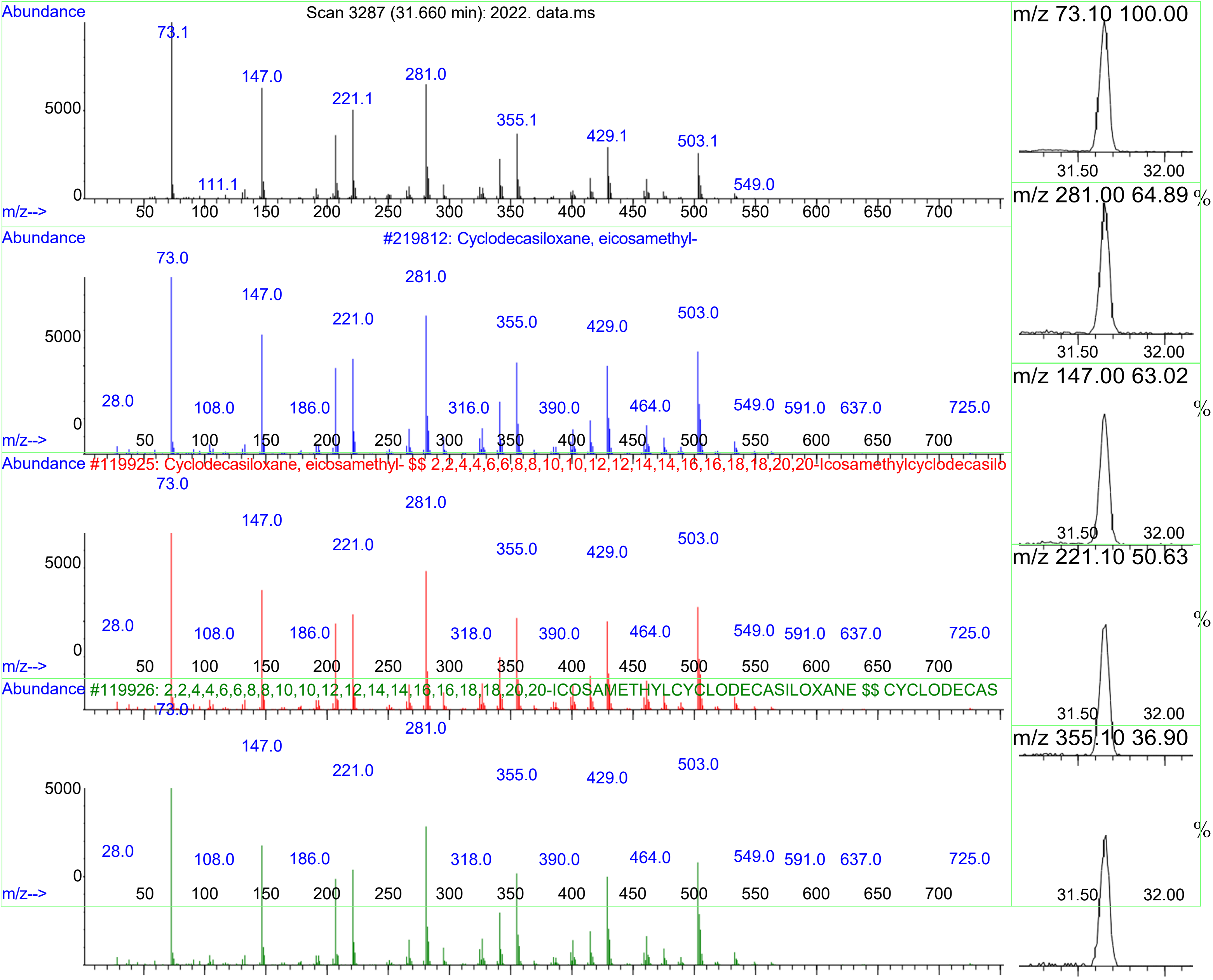
Abundance
Scan 3287 (31.660 min): 2022. data.ms
73.1
147.0
221.1
281.0
355.1
429.1
503.1
111.1
549.0
5000
0
m/z-->
50 100 150 200 250 300 350 400 450 500 550 600 650 700
Abundance
#219812: Cyclodecasiloxane, eicosamethyl-
73.0
147.0
221.0
281.0
355.0
429.0
503.0
28.0
108.0
186.0
316.0
390.0
464.0
549.0
591.0
637.0
725.0
Abundance
#119925: Cyclodecasiloxane, eicosamethyl- $$ 2,2,4,4,6,6,8,8,10,10,12,12,14,14,16,16,18,18,20,20-Icosamethylcyclodecasilo
73.0
147.0
221.0
281.0
355.0
429.0
503.0
28.0
108.0
186.0
318.0
390.0
464.0
549.0
591.0
637.0
725.0
Abundance
#119926: 2,2,4,4,6,6,8,8,10,10,12,12,14,14,16,16,18,18,20,20-ICOSAMETHYLCYCLODECASILOXANE $$ CYCLODECAS
73.0
147.0
221.0
281.0
355.0
429.0
503.0
28.0
108.0
186.0
318.0
390.0
464.0
549.0
591.0
637.0
725.0
m/z 73.10 100.00
m/z 281.00 64.89
m/z 147.00 63.02
m/z 221.10 50.63
m/z 355.10 36.90
%
31.50
32.00

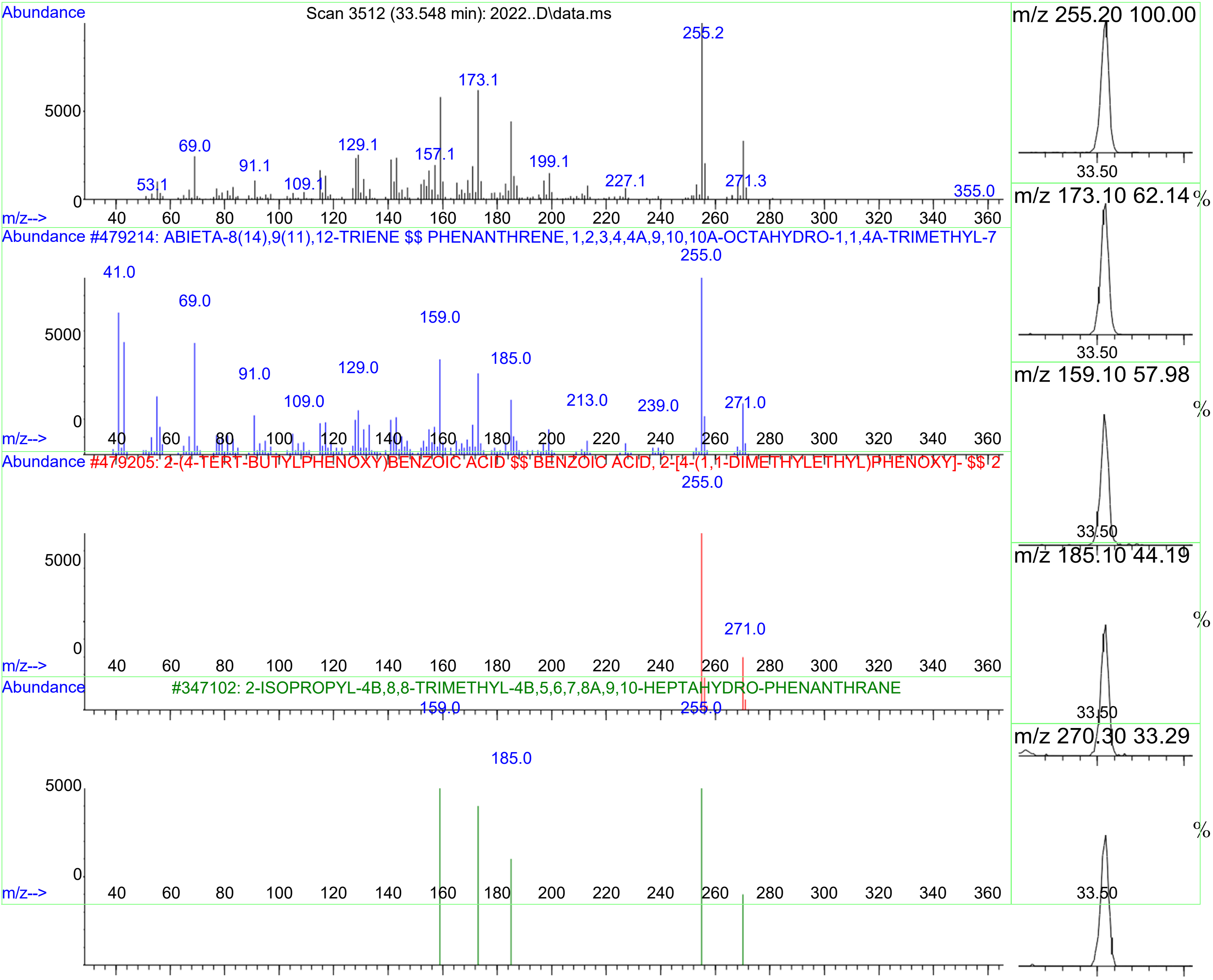
Abundance
Scan 3512 (33.548 min): 2022..D\data.ms
m/z 255.20 100.00
m/z 173.10 62.14
m/z 159.10 57.98
m/z 185.10 44.19
m/z 270.30 33.29
Abundance #479214: ABIETA-8(14),9(11),12-TRIENE $$ PHENANTHRENE, 1,2,3,4,4A,9,10,10A-OCTAHYDRO-1,1,4A-TRIMETHYL-7
Abundance #479205: 2-(4-TERT-BUTYLPHENOXY)BENZOIC ACID $$ BENZOIC ACID, 2-[4-(1,1-DIMETHYLETHYL)PHENOXY]- $$ 2
Abundance #347102: 2-ISOPROPYL-4B,8,8-TRIMETHYL-4B,5,6,7,8A,9,10-HEPTAHYDRO-PHENANTHRANE
m/z-->

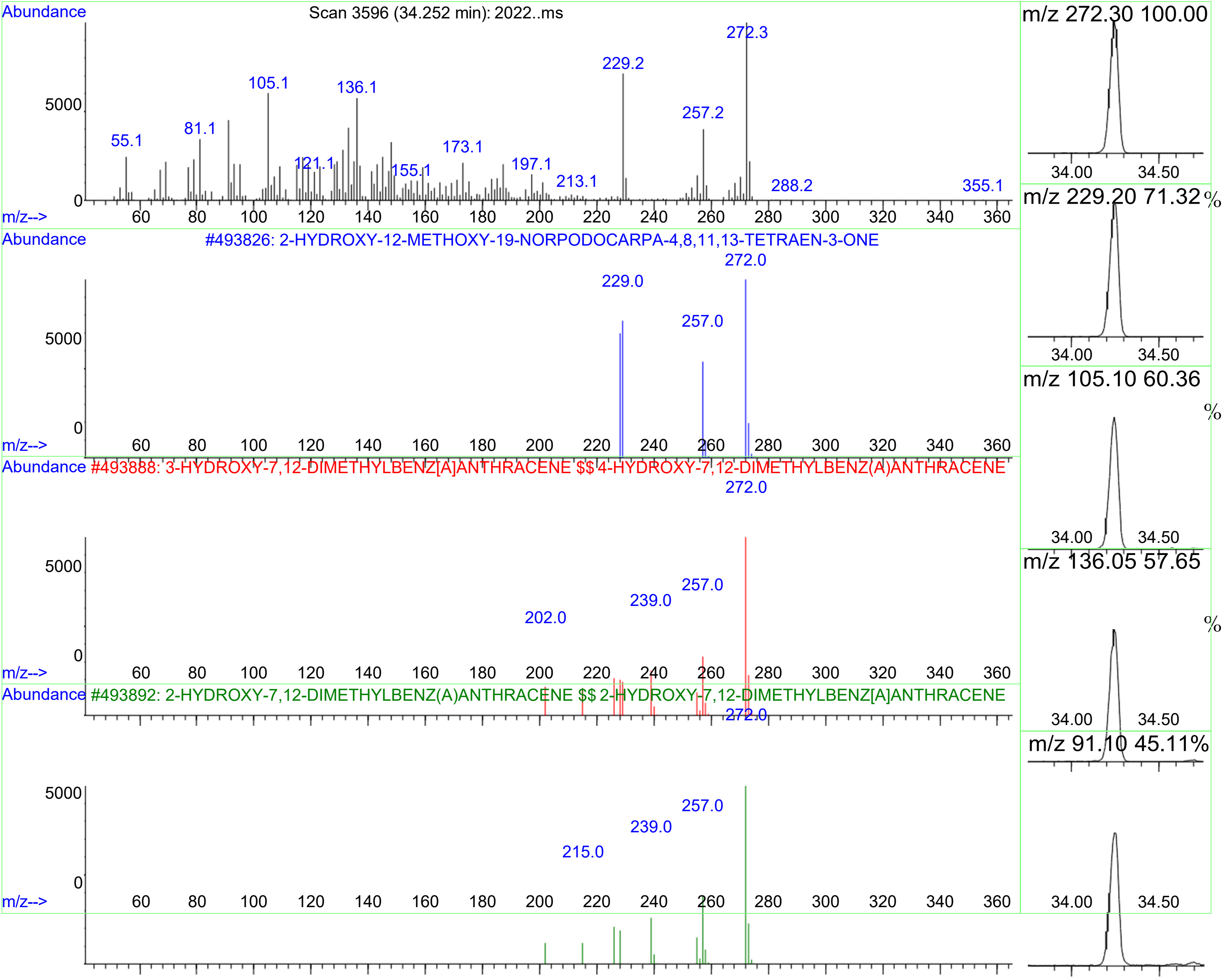
Abundance
Scan 3596 (34.252 min): 2022..ms
272.3
229.2
105.1
136.1
257.2
81.1
55.1
173.1
121.1
155.1
197.1
213.1
288.2
355.1
m/z-->
Abundance
#493826: 2-HYDROXY-12-METHOXY-19-NORPODOCARPA-4,8,11,13-TETRAEN-3-ONE
272.0
229.0
257.0
Abundance
#493888: 3-HYDROXY-7,12-DIMETHYLBENZ[A]ANTHRACENE $$ 4-HYDROXY-7,12-DIMETHYLBENZ(A)ANTHRACENE
272.0
257.0
239.0
202.0
Abundance
#493892: 2-HYDROXY-7,12-DIMETHYLBENZ(A)ANTHRACENE $$ 2-HYDROXY-7,12-DIMETHYLBENZ[A]ANTHRACENE
272.0
257.0
239.0
215.0
m/z 272.30 100.00
m/z 229.20 71.32%
m/z 105.10 60.36%
m/z 136.05 57.65%
m/z 91.10 45.11%
34.00
34.50

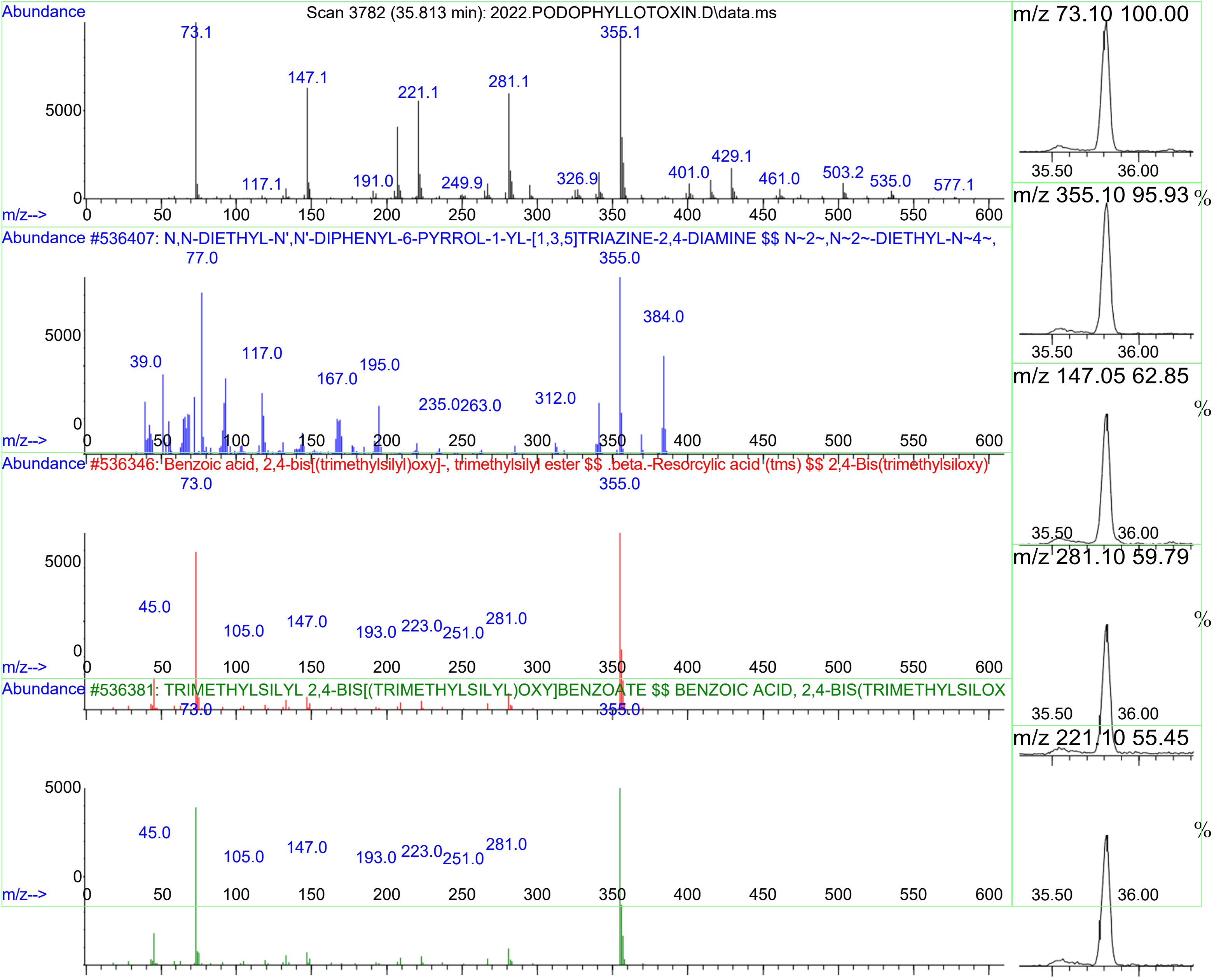
Abundance
Scan 3782 (35.813 min): 2022.PODOPHYLLOTOXIN.D\data.ms
m/z-->
Abundance #536407: N,N-DIETHYL-N',N'-DIPHENYL-6-PYRROL-1-YL-[1,3,5]TRIAZINE-2,4-DIAMINE $$ N~2~,N~2~-DIETHYL-N~4~,
Abundance #536346: Benzoic acid, 2,4-bis[(trimethylsilyl)oxy]-, trimethylsilyl ester $$ .beta.-Resorcylic acid (tms) $$ 2,4-Bis(trimethylsiloxy)
Abundance #536381: TRIMETHYLSILYL 2,4-BIS[(TRIMETHYLSILYL)OXY]BENZOATE $$ BENZOIC ACID, 2,4-BIS(TRIMETHYLSILOX
m/z 73.10 100.00
m/z 355.10 95.93 %
m/z 147.05 62.85
m/z 281.10 59.79
m/z 221.10 55.45

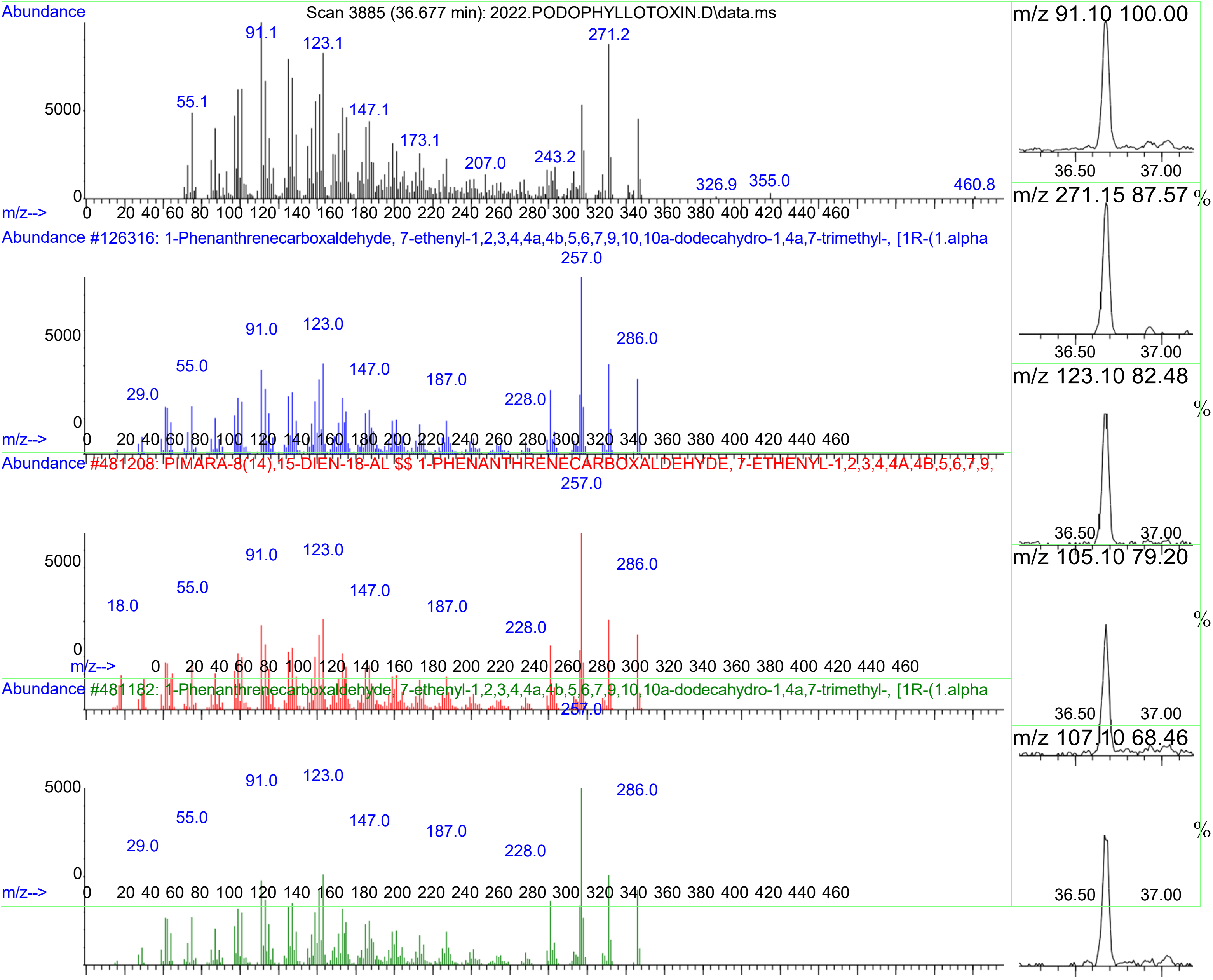
Scan 3885 (36.677 min): 2022.PODOPHYLLOTOXIN.D\data.ms
Abundance
m/z-->
Abundance #126316: 1-Phenanthrenecarboxaldehyde, 7-ethenyl-1,2,3,4,4a,4b,5,6,7,9,10,10a-dodecahydro-1,4a,7-trimethyl-, [1R-(1.alpha
Abundance #481208: PIMARA-8(14),15-DIEN-18-AL $$ 1-PHENANTHRENECARBOXALDEHYDE, 7-ETHENYL-1,2,3,4,4A,4B,5,6,7,9,
Abundance #481182: 1-Phenanthrenecarboxaldehyde, 7-ethenyl-1,2,3,4,4a,4b,5,6,7,9,10,10a-dodecahydro-1,4a,7-trimethyl-, [1R-(1.alpha
m/z 91.10 100.00
m/z 271.15 87.57 %
m/z 123.10 82.48 %
m/z 105.10 79.20 %
m/z 107.10 68.46 %
36.50
37.00

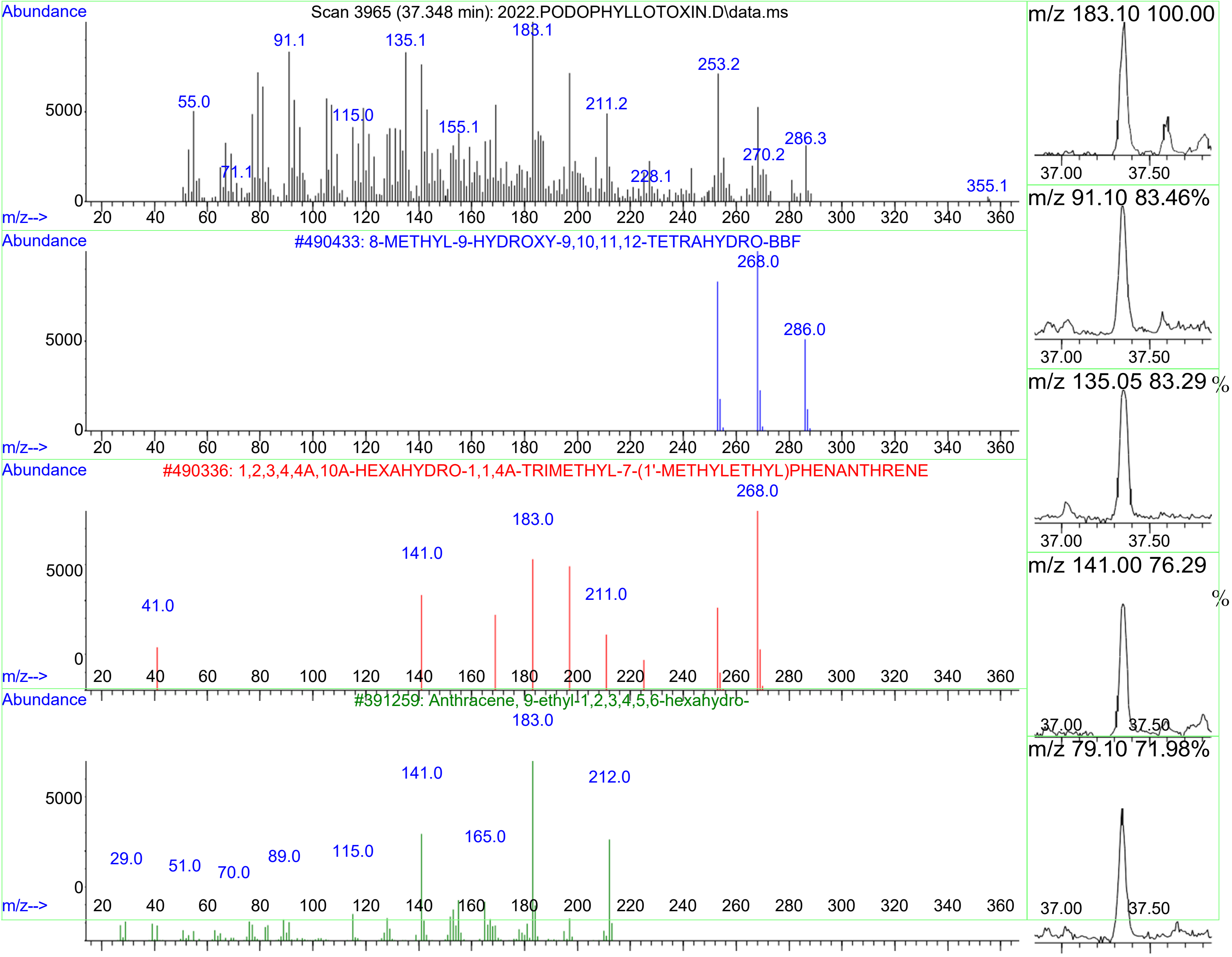
Scan 3965 (37.348 min): 2022.PODOPHYLLOTOXIN.D\data.ms
Abundance
m/z-->
55.0
71.1
91.1
115.0
135.1
155.1
183.1
211.2
228.1
253.2
270.2
286.3
355.1
#490433: 8-METHYL-9-HYDROXY-9,10,11,12-TETRAHYDRO-BBF
268.0
286.0
#490336: 1,2,3,4,4A,10A-HEXAHYDRO-1,1,4A-TRIMETHYL-7-(1'-METHYLETHYL)PHENANTHRENE
41.0
141.0
183.0
211.0
268.0
#391259: Anthracene, 9-ethyl-1,2,3,4,5,6-hexahydro-
29.0
51.0
70.0
89.0
115.0
141.0
165.0
183.0
212.0
m/z 183.10 100.00
m/z 91.10 83.46%
m/z 135.05 83.29 %
m/z 141.00 76.29 %
m/z 79.10 71.98%
37.00
37.50

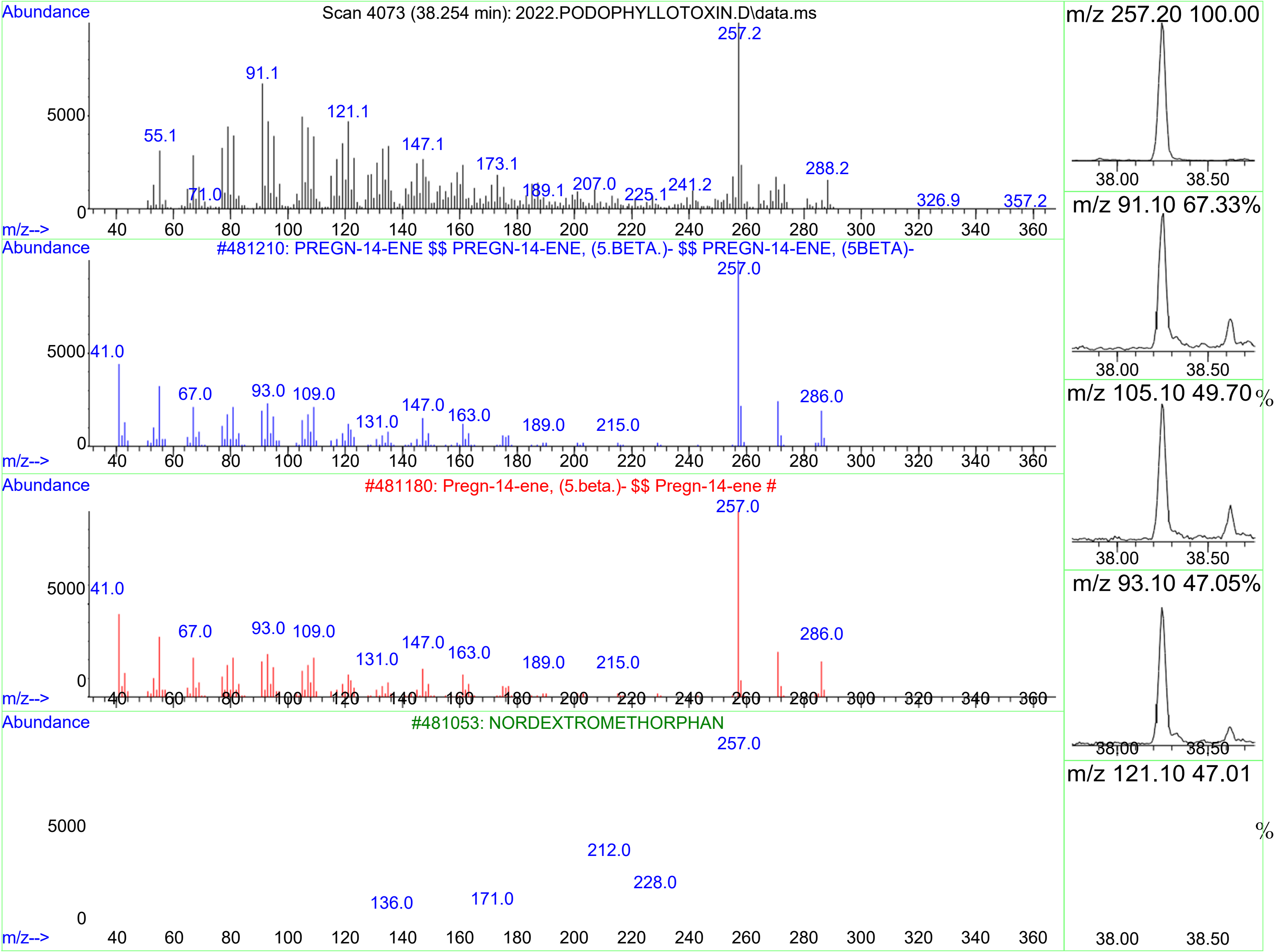
Scan 4073 (38.254 min): 2022.PODOPHYLLOTOXIN.D\data.ms
#481210: PREGN-14-ENE $$ PREGN-14-ENE, (5.BETA.)- $$ PREGN-14-ENE, (5BETA)-
#481180: Pregn-14-ene, (5.beta.)- $$ Pregn-14-ene #
#481053: NORDEXTROMETHORPHAN
Abundance
m/z-->
m/z 257.20 100.00
m/z 91.10 67.33%
m/z 105.10 49.70
m/z 93.10 47.05%
m/z 121.10 47.01

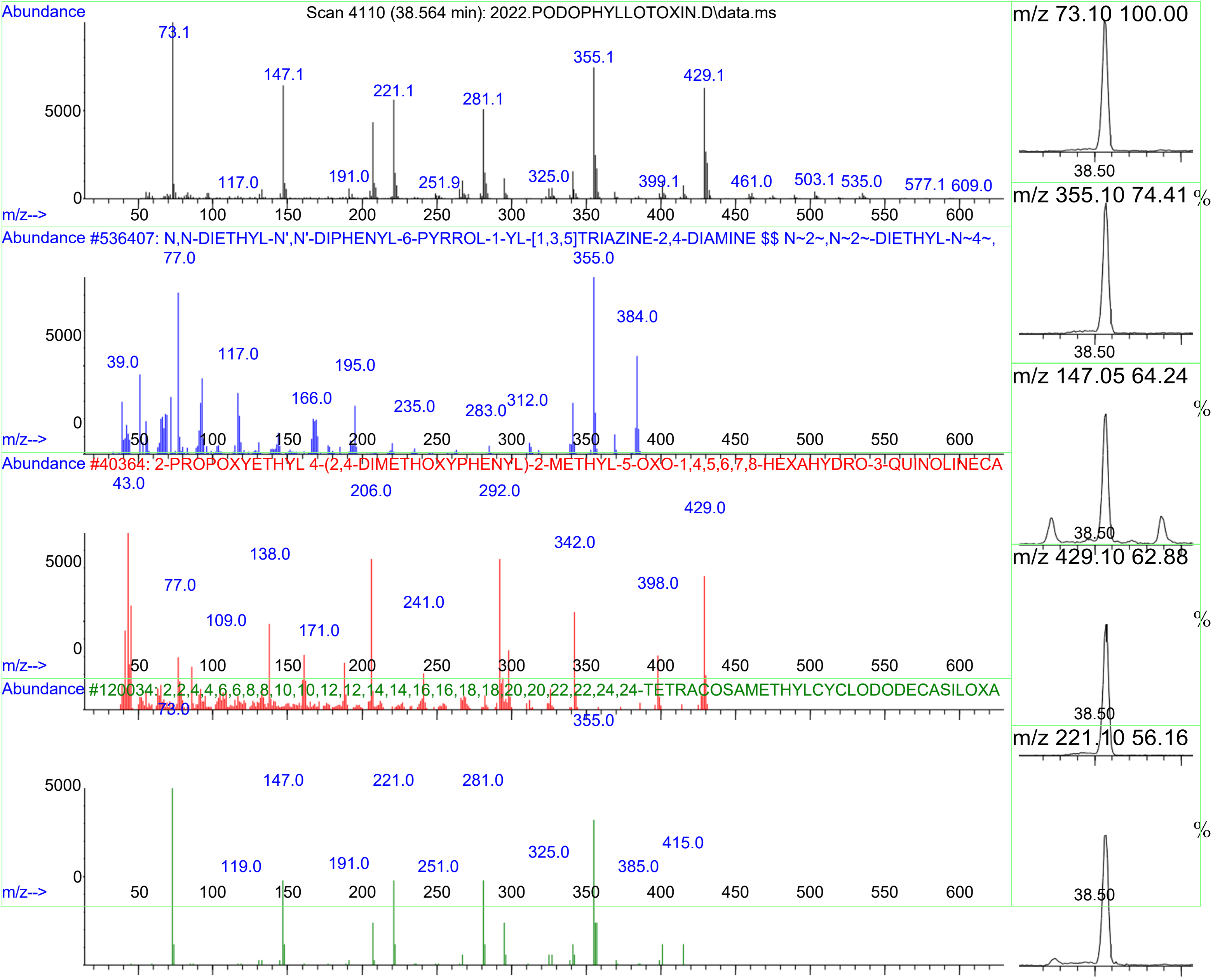
Abundance
Scan 4110 (38.564 min): 2022.PODOPHYLLOTOXIN.D\data.ms
73.1
147.1
221.1
281.1
355.1
429.1
117.0
191.0
251.9
325.0
399.1
461.0
503.1
535.0
577.1
609.0
m/z-->
Abundance #536407: N,N-DIETHYL-N',N'-DIPHENYL-6-PYRROL-1-YL-[1,3,5]TRIAZINE-2,4-DIAMINE $$ N~2~,N~2~-DIETHYL-N~4~,
77.0
355.0
384.0
39.0
117.0
195.0
166.0
235.0
283.0
312.0
Abundance #40364: 2-PROPOXYETHYL 4-(2,4-DIMETHOXYPHENYL)-2-METHYL-5-OXO-1,4,5,6,7,8-HEXAHYDRO-3-QUINOLINECA
43.0
206.0
292.0
429.0
342.0
138.0
77.0
398.0
241.0
109.0
171.0
Abundance #120034: 2,2,4,4,6,6,8,8,10,10,12,12,14,14,16,16,18,18,20,20,22,22,24,24-TETRACOSAMETHYLCYCLODODECASILOXA
73.0
355.0
147.0
221.0
281.0
325.0
415.0
119.0
191.0
251.0
385.0
m/z 73.10 100.00
m/z 355.10 74.41
m/z 147.05 64.24
m/z 429.10 62.88
m/z 221.10 56.16
38.50

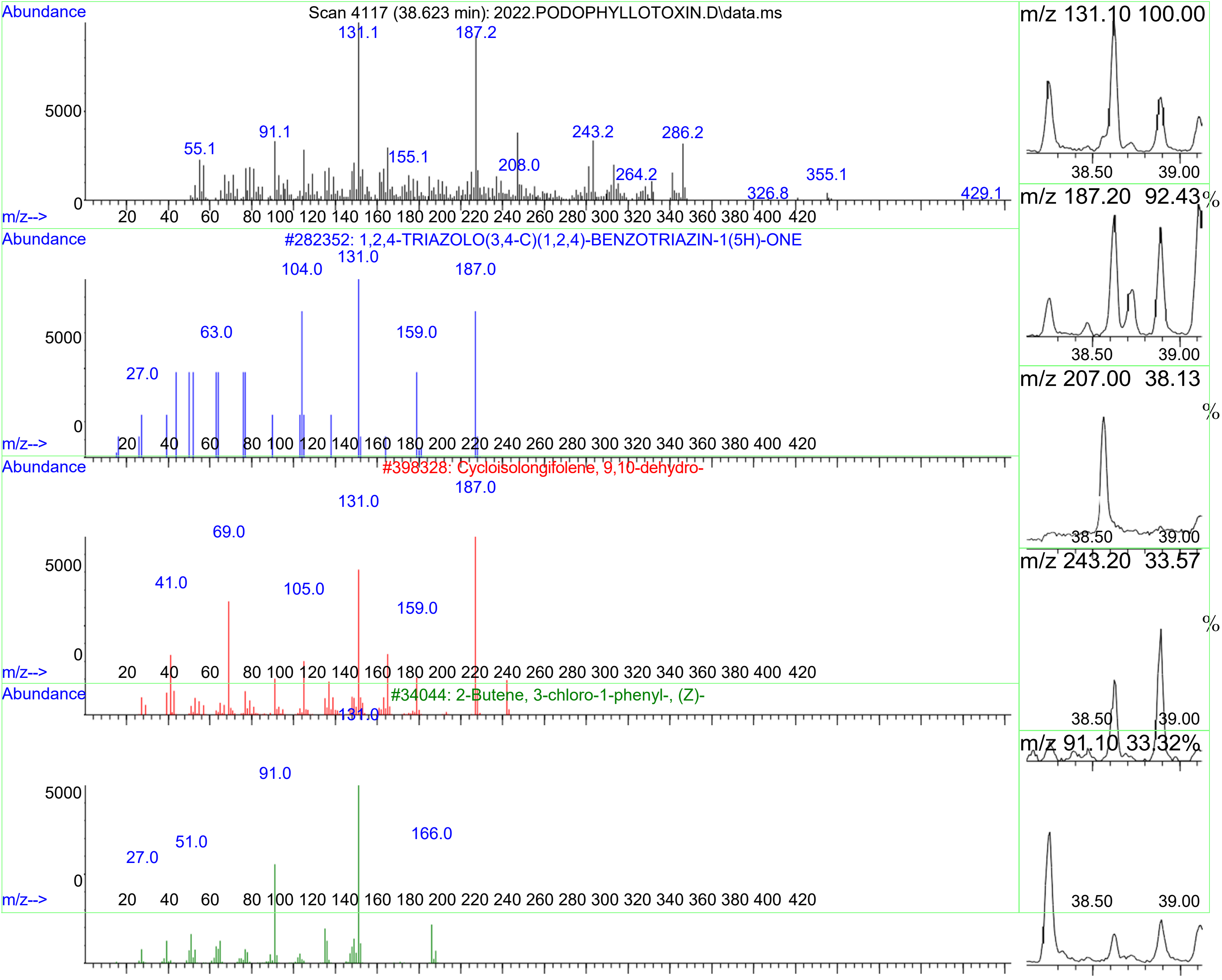
Scan 4117 (38.623 min): 2022.PODOPHYLLOTOXIN.D\data.ms
#282352: 1,2,4-TRIAZOLO(3,4-C)(1,2,4)-BENZOTRIAZIN-1(5H)-ONE
#398328: Cycloisolongifolene, 9,10-dehydro-
#34044: 2-Butene, 3-chloro-1-phenyl-, (Z)-
m/z 131.10 100.00
m/z 187.20 92.43%
m/z 207.00 38.13
m/z 243.20 33.57
m/z 91.10 33.32%
Abundance
m/z-->

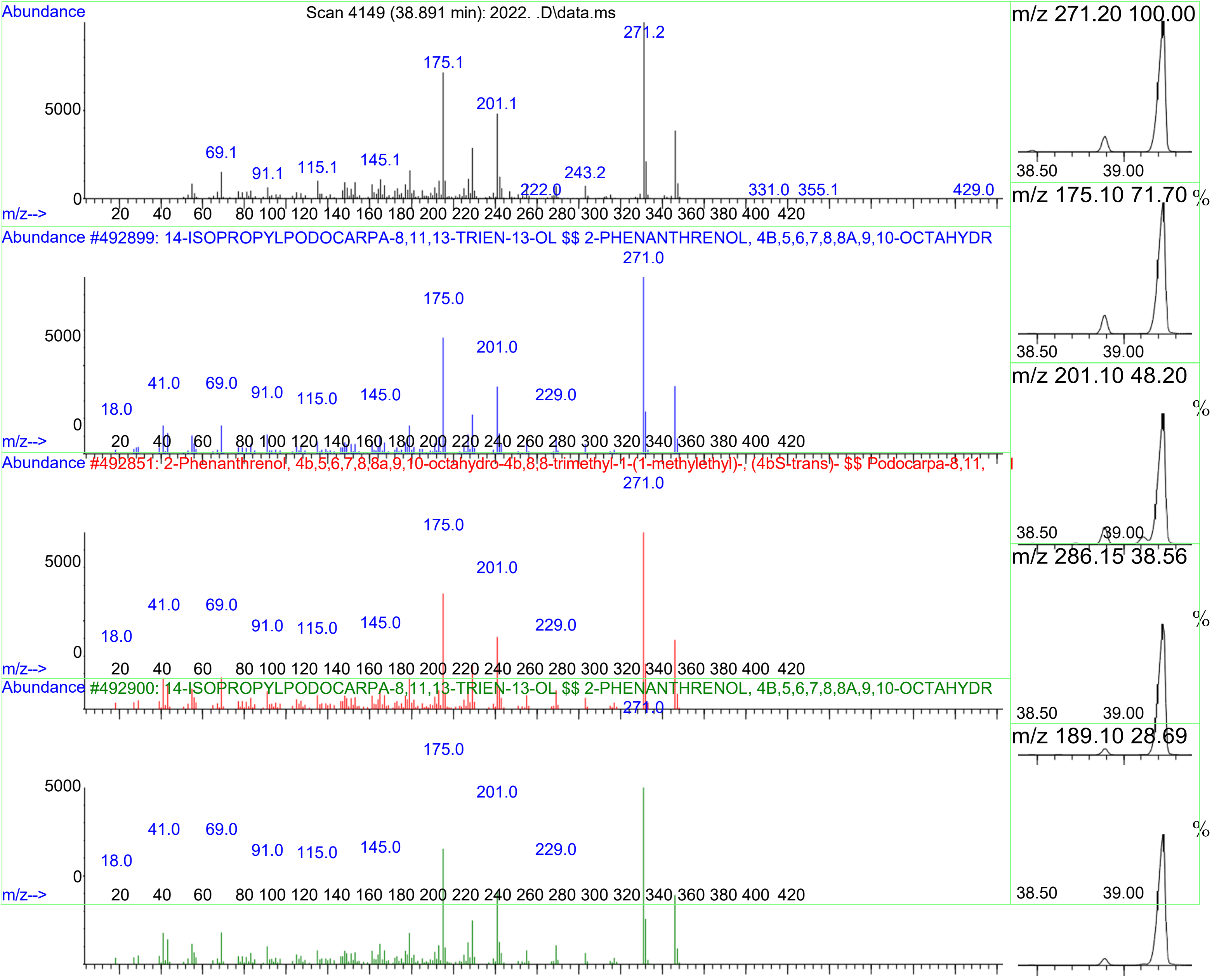
Abundance
Scan 4149 (38.891 min): 2022. .D\data.ms
271.2
175.1
201.1
69.1
91.1
115.1
145.1
243.2
222.0
331.0
355.1
429.0
5000
0
m/z-->
20 40 60 80 100 120 140 160 180 200 220 240 260 280 300 320 340 360 380 400 420
Abundance #492899: 14-ISOPROPYLPODOCARPA-8,11,13-TRIEN-13-OL $$ 2-PHENANTHRENOL, 4B,5,6,7,8,8A,9,10-OCTAHYDR
271.0
175.0
201.0
41.0
69.0
91.0
115.0
145.0
229.0
18.0
Abundance #492851: 2-Phenanthrenol, 4b,5,6,7,8,8a,9,10-octahydro-4b,8,8-trimethyl-1-(1-methylethyl)-, (4bS-trans)- $$ Podocarpa-8,11,
271.0
175.0
201.0
41.0
69.0
91.0
115.0
145.0
229.0
18.0
Abundance #492900: 14-ISOPROPYLPODOCARPA-8,11,13-TRIEN-13-OL $$ 2-PHENANTHRENOL, 4B,5,6,7,8,8A,9,10-OCTAHYDR
271.0
175.0
201.0
41.0
69.0
91.0
115.0
145.0
229.0
18.0
m/z 271.20 100.00
m/z 175.10 71.70 %
m/z 201.10 48.20 %
m/z 286.15 38.56 %
m/z 189.10 28.69 %
38.50
39.00

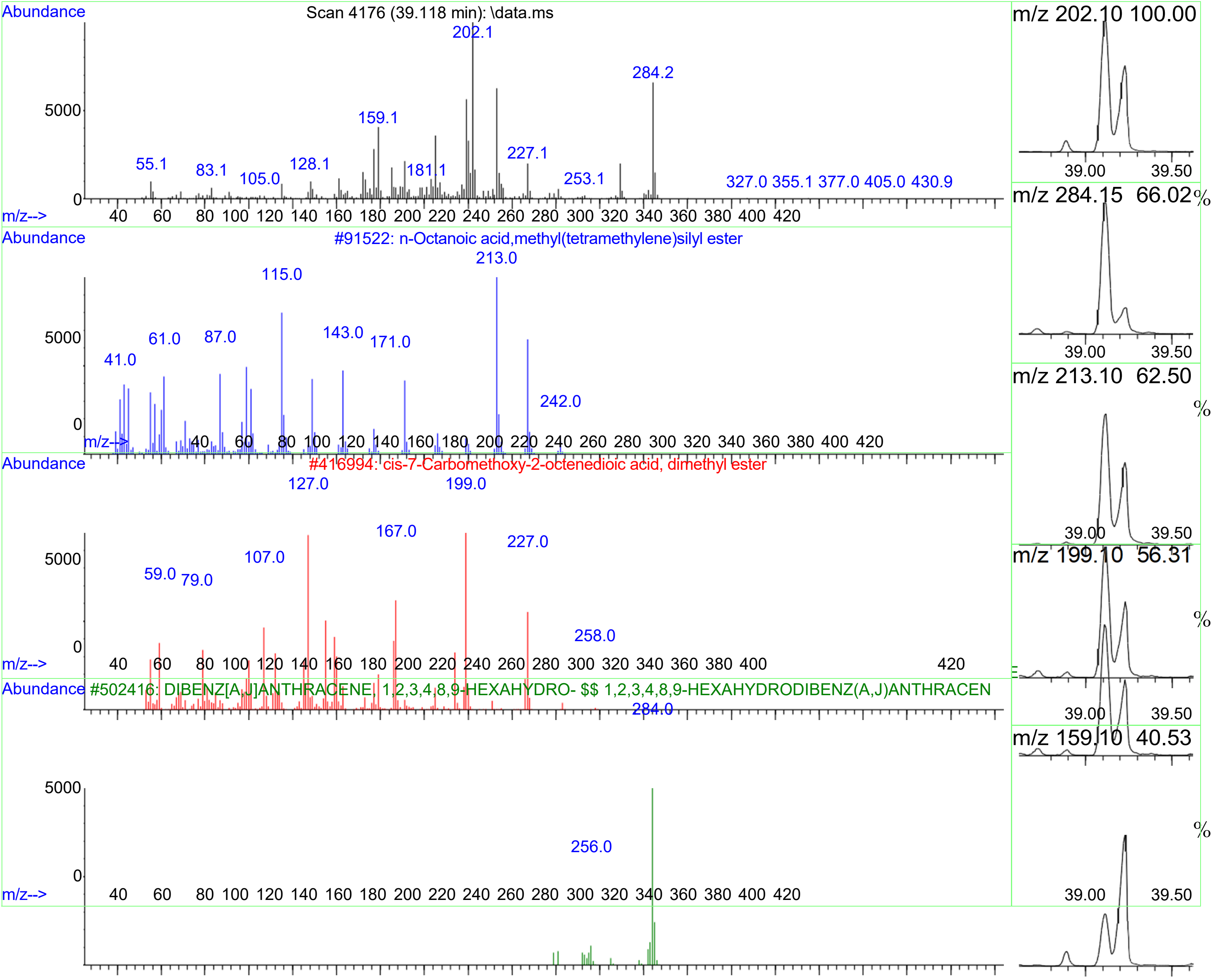

Abundance
Scan 4176 (39.118 min): \data.ms
202.1
284.2
159.1
55.1
83.1
105.0
128.1
181.1
227.1
253.1
327.0 355.1 377.0 405.0 430.9
m/z-->
#91522: n-Octanoic acid,methyl(tetramethylene)silyl ester
213.0
115.0
61.0
87.0
143.0
171.0
41.0
242.0
#416994: cis-7-Carbomethoxy-2-octenedioic acid, dimethyl ester
127.0
199.0
167.0
227.0
107.0
59.0
79.0
258.0
#502416: DIBENZ[A,J]ANTHRACENE, 1,2,3,4,8,9-HEXAHYDRO- $$ 1,2,3,4,8,9-HEXAHYDRODIBENZ(A,J)ANTHRACEN
284.0
256.0
m/z 202.10 100.00
m/z 284.15 66.02
m/z 213.10 62.50
m/z 199.10 56.31
m/z 159.10 40.53
39.00
39.50

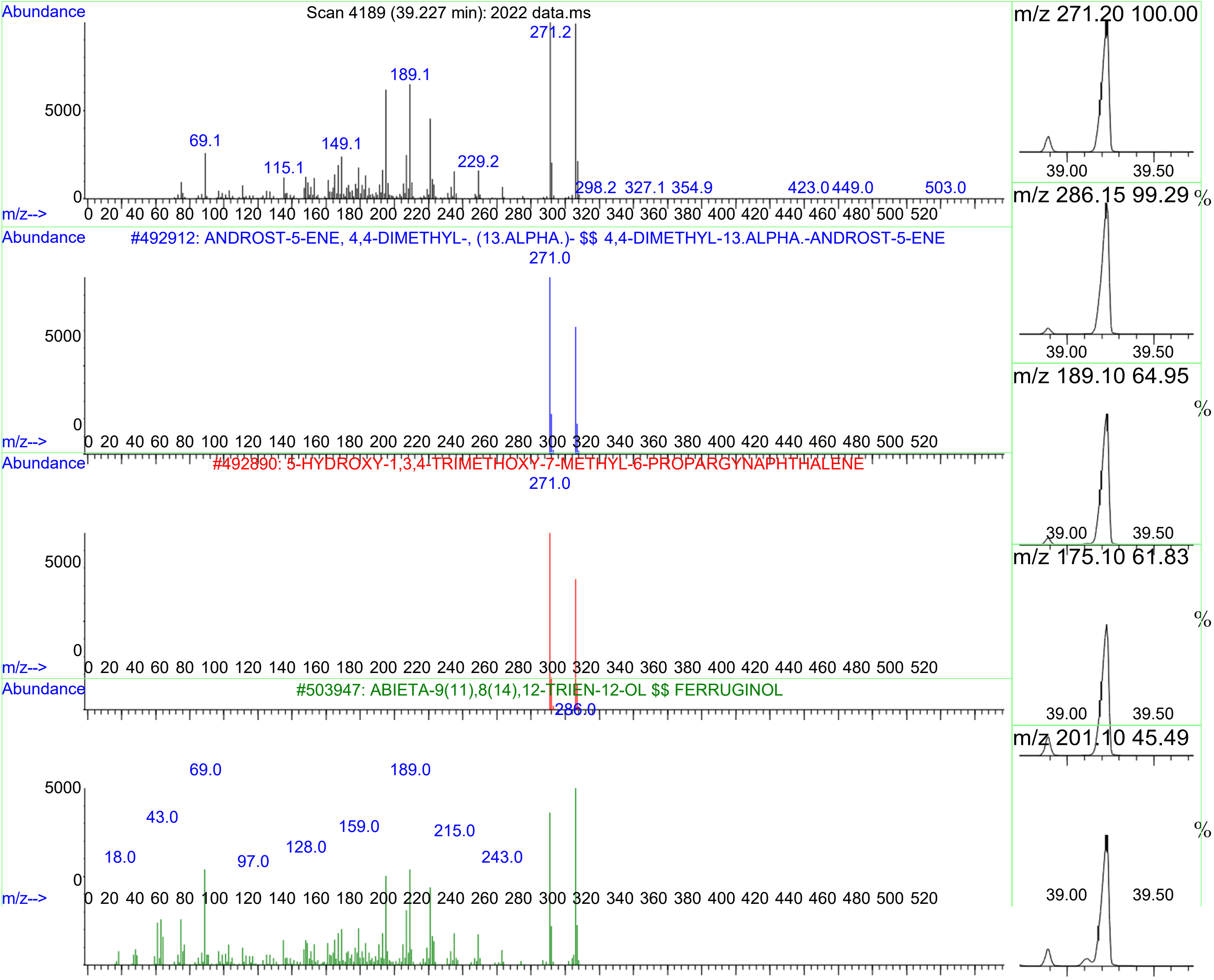
Abundance
Scan 4189 (39.227 min): 2022 data.ms
271.2
189.1
69.1
149.1
115.1
229.2
298.2 327.1 354.9
423.0 449.0
503.0
m/z-->
m/z 271.20 100.00
m/z 286.15 99.29
m/z 189.10 64.95
m/z 175.10 61.83
m/z 201.10 45.49
39.00
39.50
Abundance
#492912: ANDROST-5-ENE, 4,4-DIMETHYL-, (13.ALPHA.)- $$ 4,4-DIMETHYL-13.ALPHA.-ANDROST-5-ENE
271.0
Abundance
#492890: 5-HYDROXY-1,3,4-TRIMETHOXY-7-METHYL-6-PROPARGYNAPHTHALENE
271.0
Abundance
#503947: ABIETA-9(11),8(14),12-TRIEN-12-OL $$ FERRUGINOL
286.0
69.0
189.0
43.0
159.0
215.0
18.0
128.0
97.0
243.0

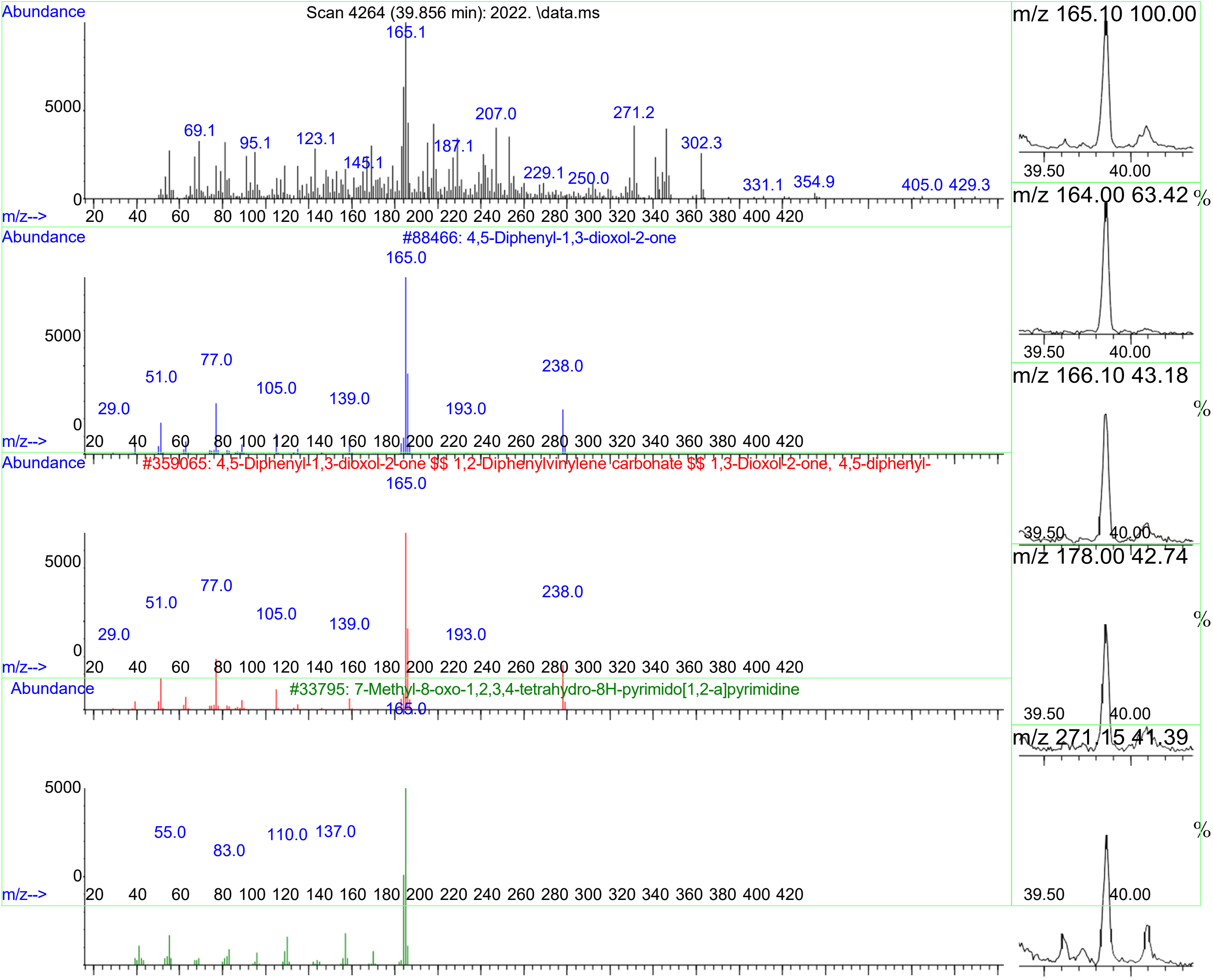
Abundance
Scan 4264 (39.856 min): 2022. \data.ms
165.1
5000
69.1
95.1
123.1
145.1
187.1
207.0
229.1
250.0
271.2
302.3
331.1
354.9
405.0
429.3
m/z-->
m/z 165.10 100.00
m/z 164.00 63.42
m/z 166.10 43.18
m/z 178.00 42.74
m/z 271.15 41.39
39.50
40.00
#88466: 4,5-Diphenyl-1,3-dioxol-2-one
165.0
29.0
51.0
77.0
105.0
139.0
193.0
238.0
#359065: 4,5-Diphenyl-1,3-dioxol-2-one $$ 1,2-Diphenylvinylene carbonate $$ 1,3-Dioxol-2-one, 4,5-diphenyl-
#33795: 7-Methyl-8-oxo-1,2,3,4-tetrahydro-8H-pyrimido[1,2-a]pyrimidine
55.0
83.0
110.0
137.0
165.0

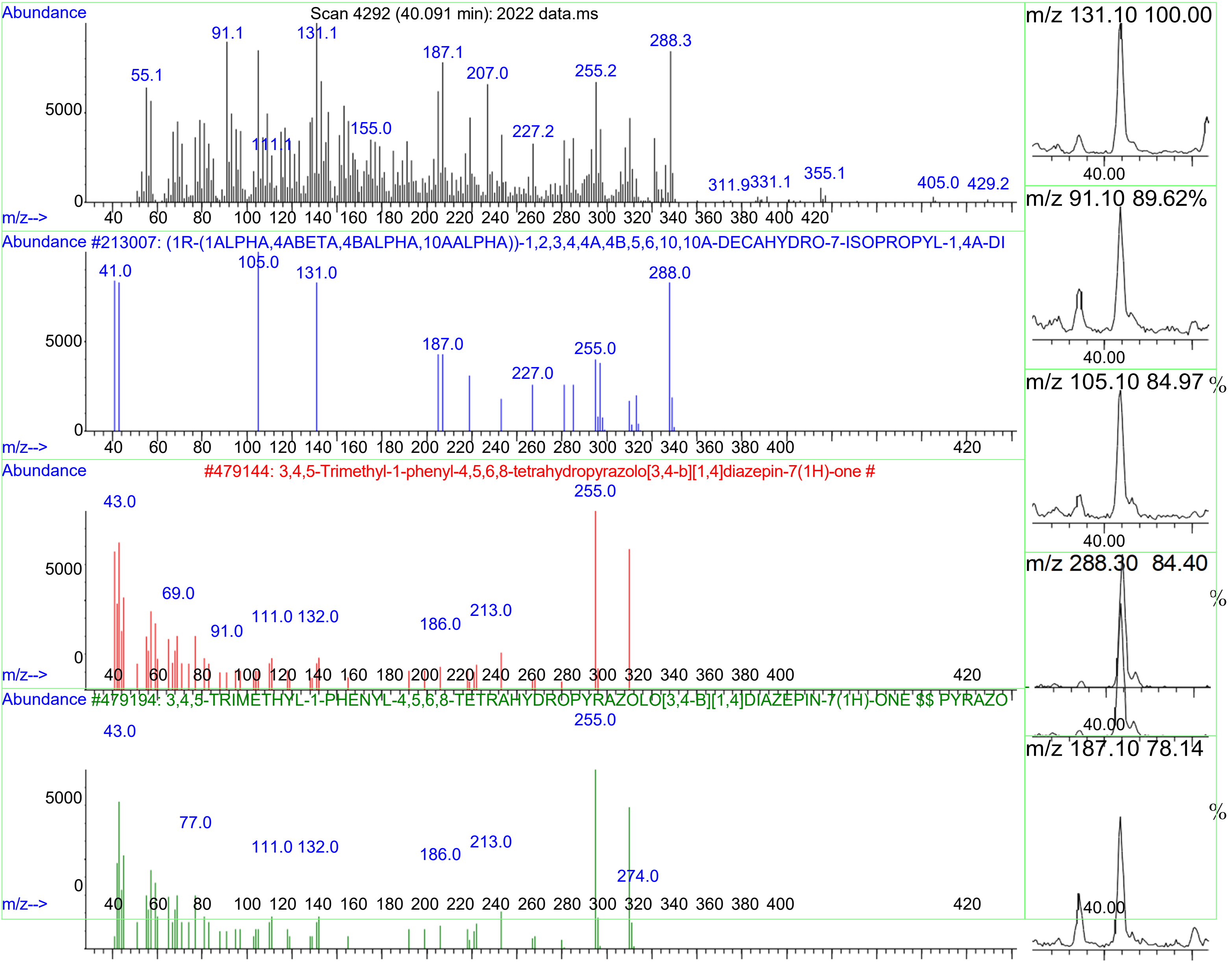

Figure S1. Mass spectrum of identified. phytochemical compounds from leaf extract of *Juniperus procera*.
